# Draft Genome Sequence of the Fish Strain *Edwardsiella tarda* NCIMB 2034

**DOI:** 10.1128/genomeA.00359-17

**Published:** 2017-05-18

**Authors:** Noemí Buján, Aide Lasa, Alicia E. Toranzo, Beatriz Magariños

**Affiliations:** Departamento de Microbioloxía y Parasitoloxía, CIBUS-Facultade de Bioloxía and Instituto de Acuicultura, Universidade de Santiago de Compostela, Santiago de Compostela, Spain

## Abstract

*Edwardsiella tarda* is an important pathogen for fish. The strain NCIMB 2034, obtained from the National Collection of Industrial Food and Marine Bacteria, was isolated from unknown diseased fish in the United States. The draft genome sequence has 3.79 Mb with a G+C content of 57.1% and >3,340 protein-coding genes.

## GENOME ANNOUNCEMENT

*Edwardsiella tarda* was the first described species of the genus *Edwardsiella* in 1965 by Ewing et al. (1). This bacterium is a Gram-negative facultative aerobic pathogen belonging to the *Enterobacteriaceae* family. *E. tarda* is widely distributed in aquatic environments and has a broad host range including fish, reptiles, amphibians, and humans (2). *E. tarda* shares many traits with the recently described *E. piscicida* (3), such as phenotypic characteristics, host range, and clinical signs, with few differences. This fact makes it almost impossible to accurately assign isolates to one or the other species. Therefore, it is necessary to use molecular techniques to properly identify *E. tarda* isolates and differentiate them from *E. piscicida*.

Genomic DNA of *E. tarda* NCIMB 2034 was extracted using a High Pure PCR template preparation kit (Roche Diagnostics) following the manufacturer’s instructions. The purified DNA genome was sequenced at Macrogen, Inc. (Seoul, Republic of Korea) using Illumina paired-end sequencing technology. Reads were trimmed and filtered to remove adapters and low-quality bases, using the Trimmomatic 0.32 (4) program. The remaining reads were used for the genome assembly, performed with the SPAdes 3.6.2 novo assembler tool (5), and QUAST (6) software was used to evaluate the assembly. The *E. tarda* genome assembly of 542 contigs (>200 bp) resulted in a genome size of 3,798,355 bp with a G+C% content of 57%.

The genome was submitted for annotation to the Rapid Annotations using Subsystems Technology (RAST) server (7). The genomic features of NCIMB 2034 included a total of 3,349 coding sequences (CDSs) and 61 possible missing genes. Using the software tRNAscan-SE v1.21 (8), 83 tRNA sequences from a total of 105 RNAs were identified.

According to the annotation results, the genome revealed the presence of genes coding for resistance to antibiotics and toxic compounds, beta-lactamases, copper homeostasis, cobalt-zinc-cadmium resistance, resistance to fluoroquinolones, arsenic and mercury resistance, and multidrug resistance efflux pump subsystems. Moreover, the presence of the genes *bar*A and *sir*A, systems that increase the expression of virulence genes and decrease the expression of motility genes, were detected. The genomic region that contains the *yid*E gene, coding for a mediator on hyperadherence in the family *Enterobacteriaceae*, was found. In addition, 134 coding sequences related to stress responses, such as osmotic and oxidative stress, cold and heat shock stress responses, and genes involved in the uptake of selenium oxyanions for biological detoxification, were detected.

### Accession number(s).

This whole-genome shotgun project has been deposited at DDBJ/EMBL/GenBank under the accession no. MSSL00000000. The version described in this paper is version MSSL01000000.
